# Safety of Intravitreal Injection of Biosimilar of Aflibercept in Rabbit Eyes

**DOI:** 10.1155/2020/2602918

**Published:** 2020-07-03

**Authors:** Alireza Lashay, Hamid Riazi-Esfahani, Hooshang Faghihi, Ahmad Mirshahi, Hassan Khojasteh, Alireza Khodabande, Fahimeh Asadi Amoli, Fariba Ghassemi, Fatemeh Bazvand, Elias Khalili Pour, Nazanin Ebrahimiadib, Ali Torkashvand, Elham Delrish

**Affiliations:** Eye Research Center, Farabi Eye Hospital, Tehran University of Medical Sciences, Tehran, Iran

## Abstract

**Purpose:**

To assess the safety of biosimilar intravitreal aflibercept (CinnaGen Co., Iran) compared to the reference product (Eylea®; Bayer Schweiz AG, Zurich, Switzerland) in rabbit eyes through functional and histologic studies.

**Methods:**

Forty New Zealand albino rabbits were recruited to the study and were divided into four groups to be sacrificed at 48 hours, one, two, and four weeks after injections. In each group, five rabbits received 0.05 mL (2 mg) biosimilar aflibercept in the right eye and 0.05 mL saline in the left eye as the control, and in a similar manner, the remaining five rabbits received the reference drug in the right eye and saline in the left eye. All the rabbits underwent comprehensive ophthalmic examination and electroretinography (ERG) tests at baseline and also just before enucleation at the specific predefined time points. The enucleated eyes were prepared for retinal toxicity histological examination.

**Results:**

No retinal toxicity was observed based on histologic and ERG findings in all groups. Choroidal congestion was revealed after 1 week in an eye that was injected with biosimilar aflibercept, although the similar finding was detected in the contralateral eye which received saline. Also, one subject which received the reference drug showed chronic vitritis and lymphoplasmocytic reaction of the optic disc at week 4. The remaining subjects showed no histologic changes.

**Conclusion:**

The 2 mg intravitreal injection of biosimilar aflibercept (CinnaGen Co., Iran) was found to be nontoxic in rabbit eyes in the short-term period. Further studies are required to warrant the efficacy and safety profile of the drug in human subjects.

## 1. Introduction

In 1989, the VEGF was introduced as a protein responsible for vascular permeability and endothelial cell proliferation that plays a key role in angiogenesis. Thereafter, it became the core of the treatment of ophthalmic vascular and degenerative disorders [1, 2].

Previously, level of intravitreal VEGF has been revealed to be correlated with the severity of the retinal vascular diseases, and inhibition of this protein in experimental models has been shown to regress the new vessels of the retina and choroid and prevent visual impairment [1, 3].

Aflibercept under the brand name of Eylea^®^ (Bayer Schweiz AG, Zurich, Switzerland) received FDA approval for the treatment of neovascular age-related macular degeneration (nAMD) and diabetic macular edema (DME) [4]. It consists of VEGF receptor1, 2-FC, a fusion protein which blocks VEGFs A and B and placental growth factor [5].

As many of these patients need frequent intravitreal injections, one arising issue, in this regard, is the treatment burdens imposed on the health care systems [4, 6].

Nowadays, many biosimilar agents have been commercially available. They are exactly analogous to the reference drugs in terms of structure, safety, and efficacy but with different production methods and lower cost relative to the reference drugs, which facilitate treatment access particularly in the developing countries [7, 8].

This study was conducted to determine the safety and possible toxicity of the intravitreal injection of the biosimilar aflibercept (CinnaGen Co., Iran) in albino rabbits in terms of histopathologic and functional evaluations.

## 2. Methods

Forty New Zealand albino rabbits, between 1.5 and 2.5 kg, were recruited to assess the safety of intravitreal biosimilar aflibercept injection. All procedures were performed following the principal tenets of using animals in ophthalmic and vision research (ARVO). In addition, approval from Tehran University of Medical Science's Institutional Review Board (IRB) and ethics committee was obtained.

The rabbits were kept in an air-conditioned room with a 12-hour light-dark cycle and fed with standard processed laboratory food.

Four groups of the rabbits (10 in each groups) were planned for the injections. Ten rabbits were programmed or assigned to be sacrificed 48 hours after injection, and the remaining were divided into three separate groups to be sacrificed one (10 rabbits), two (10 rabbits), and four weeks (10 rabbits) after injections. Clinical examinations and electroretinogram (ERG) were performed at baseline and just before sacrificing the rabbits. Five rabbits of each group were randomly selected to receive biosimilar aflibercept in their right eye and saline in the left, and the remaining five rabbits in each group received the reference drug (Eylea^®^) in their right eye and saline in the left.

Any rabbits with documented anterior or posterior segment abnormalities were excluded from the study. The rabbits were anesthetized before all procedures using ketamine hydrochloride (50 mg/kg) and xylazine hydrochloride (5 mg/kg). Eye examinations were done through the dilated pupil by topical tropicamide (0.5%) eye drop at each session.

### 2.1. Drug Preparations and Injections

The rabbits were anesthetized with the aforementioned combination of drugs. They underwent baseline ophthalmic examination and ERG, and then the injection was performed under a sterile condition using a 29-gauge needle after instillation of povidone-iodine 5% into fornixes for 2 minutes.

All injections were performed through 1.5 mm posterior to the limbus into the midvitreous. Chloramphenicol and timolol eye drops were applied to the eyes for the first three days after injections.

### 2.2. Clinical Observations

All the eyes underwent thorough ophthalmic examination at baseline, first and second days following injections. Furthermore, the examinations were performed at weeks one, two, and four after injection using the hand-held slit-lamp and indirect ophthalmoscope before considering the eyes for enucleation.

Anterior chamber and vitreous cavity were carefully examined with the highest magnification to detect any cell or flare. At each follow-up visit, all the eyes underwent indirect ophthalmoscopy to be assured of sharp and clear imaging of the disc and the retina and to exclude any pathologic finding in these structures.

Any conjunctival injection, corneal opacity, or ulcer, crystalline lens damage or abnormality, inflammatory responses in the anterior chamber or the vitreous cavity, and retinal pathology were recorded.

### 2.3. Electrophysiology

All the rabbits underwent ERG, using the electrophysiological test system (Metrovision, France), at baseline and days 2, 7, 14, and 28 following injections regarding their groups. Before the test, all dark-adapted rabbits were anesthetized, and ERG was performed by means of a contact lens on the cornea, a negative electrode, and ground electrode that were placed at the orbital rim and ear, respectively. The intensity of the flash light was selected 10 cdsm [2], and response of eight repeated stimulations was averaged as response. Amplification (320,000) of the filtered signals (0.3–300 Hz) was applied by differential amplifiers. Then, the amplitude and implicate time of the *a*- and *b*-wave were measured as ERG responses. The *a*-wave amplitude was defined as the distance from the isoelectric line to the negative spike and the *b*-wave as the distance from the negative spike to the positive spike. Latencies of the *a* and *b* waves were measured form the time of presenting the stimuli.

### 2.4. Histopathologic Examinations

At the final visit, all rabbits underwent ophthalmic examination and ERG under deep anaesthesia, and then they were euthanized using 100 mg/kg of sodium pentobarbital intravenous injection, and the eyes were enucleated with cautions to prevent probable globe violation. All eyes were transferred into a neutral formalin medium as soon as possible and then fixed by 10% formalin to prevent tissue degradation.

Tissue processing and specimen preparation were performed by a pathologist well experienced in ophthalmic diseases, and a section of the tissue with 4 *µ*m thickness was prepared and stained with hematoxylin and eosin (H&E).

We performed the mentioned process for almost all parts of the eye including conjunctiva, cornea, iris, ciliary body, lens, choroid, retina, sclera, optic disc, anterior chamber, and angle vitreous by means of a light microscope.

Any abnormal finding including edema, haemorrhage, inflammatory responses, necrosis, and deposition of the unusual materials was considered. Also, different retinal layers' thickness, number of the ganglion cells, and changes of the retinal pigment epithelium (RPE) layers were taken into account.

## 3. Statistical Analysis

The Mann–Whitney U-test was used to analyse the effect of the injections on the ERG parameters between the biosimilar aflibercept and the reference drug (Eylea^®^) groups and also between the biosimilar aflibercept and the saline groups, separately. The changes more than 20% of the baseline in the ERG parameters were considered significant in each group. All data were analyzed using SPSS (version. 22.0; SPSS, Chicago, IL), and the significance value of *P* was considered less than 0.05.

## 4. Results

From 40 rabbits which were enrolled in the study, 38 rabbits completed the trial. One rabbit in each group of biosimilar aflibercept and Eylea^®^ died and was excluded from the study. The reaming well tolerated the drugs, and there was no obvious alteration in behaviour of the animals in terms of feeding and mobility.

### 4.1. Clinical Evaluation

Anterior segment examination revealed no significant abnormality in the conjunctiva or different layers of the cornea. Anterior chamber or vitreous cell was not detected in any eye at different time points on biomicroscopic examination. There was no iris abnormality in any eye of each group. The crystalline lenses were significantly clear. The vitreous, retina, choroid, and optic nerve seemed normal based on indirect ophthalmoscopy.

### 4.2. Electrophysiology

At baseline, there was no significant difference between the biosimilar aflibercept, Eylea, and saline groups based on ERG parameters, neither amplitude nor implicit time of the *a* or *b* waves (*P* > 0.5). These ERG parameters showed no significant difference in each time point after injections between the biosimilar aflibercept-injected eyes and the control groups (Eylea^®^ and saline) (Table 1).

As aforementioned, the changes of ERG parameters more than 20% were considered significant during the study. Tables 2 and 3 show the percentage of the changes at each time point relative to the baseline ERG in the biosimilar aflibercept-injected group and the reference drug-injected group, respectively: there was no remarkable change in the ERG parameters in both groups comparing to the baseline.

Dark-adapted bright flash ERG was done on all rabbits before the intravitreal injection as a baseline standard and then at each time point. Despite of a 14.6% reduction of *a*-wave amplitude at day 14 in the biosimilar group compared with the baseline, fairly comparable to the Eylea arm that was 10.5% at week 4, no significant changes were found in *a*-wave amplitude and implicit time after injections in each group.

Similarly, there were no clinically significant changes in *b*-wave amplitude and latency during the follow-up visits of the subjects. There was a comparable reduction of *b*-wave amplitude in biosimilar aflibercept and the reference drug-injected eyes (9.1% and 10%, respectively) at week 4.

### 4.3. Histological Findings

Considering presence of chronic conjunctivitis in some specimens from both control and drug-injected groups, this pathological finding is not necessarily related to drug administration.

All the eyes which were enucleated 2 days after biosimilar aflibercept injection had no pathologic finding unless one that showed mild chronic conjunctivitis and iridocyclitis; similar finding was evident in the contralateral eye which received saline.

Among rabbits that were enucleated one week after injections, mild choroidal congestion was reported in both eyes of a rabbit that received biosimilar aflibercept and normal saline into the right and left eye, respectively. Chronic vitritis and lymphoplasmocytic infiltration of the optic nerve head were detected at 4 weeks after injection in an eye that was injected with the reference drug (Eylea^®^).

Except the findings mentioned above, there was no distinguishable change in the biosimilar aflibercept, Eylea^®^, and saline control eyes after intravitreal injections based on histopathologic evaluations.

## 5. Discussion

Our results showed that a single intravitreal injection of biosimilar aflibercept at doses up to 2 mg in albino rabbits' eyes did not result in apparent vitreoretinal toxicity at 2, 7, 14, and 28 days after injection based on electrophysiological and histopathological findings. The ERG responses of the experimental and two control eye groups were similar in *a*- or *b*-wave amplitude and implicit time at different time points after injections. Despite the variability of ERG responses during the study that may be influenced by various factors including the depth of sedation and technical bias, no significant change in the ERG parameter beyond our predefined threshold (20%) was detected (Tables 2 and 3).

Currently, anti-VEGFs have a key role in the management of various retinal conditions, particularly vascular diseases like nAMD, diabetic retinopathy, and retinal vein occlusions [5, 9, 10]. Several studies evaluate the efficacy and safety of aflibercept in comparison to the previous anti-VEGF drugs [11]. Some studies showed its superiority in terms of visual gain, retinal thickness, and number of injections in particular conditions [11, 12]. Diabetic Retinopathy Clinical Research Network (DRCR.net) Protocol T revealed the similar outcomes of aflibercept compared to bevacizumab and ranibizumab, further the superiority of aflibercept over bevacizumab among eyes with baseline visual acuity of 20/50 to 20/320 [13]. The PLANET study also showed that monotherapy with aflibercept is an acceptable choice for the management of polypoidal choroidal vasculopathy (PCV) [14]. Nonetheless, the big problem is surrounding the costs. Based on the 2015 wholesale, one injection costs $1,850 for 2 mg aflibercept, which was roughly 31 times costlier than bevacizumab 1.25 mg [15, 16]. Moreover, most of the patients need frequent and multiple injections that induce high individual and public costs [15]. Since one of the important factors that restricts the access to treatment particularly in developing countries is high financial burden, by decreasing the treatment burdens, improvement in patient's adherence to the treatment would be possible [17]. In this regard, some similar drugs to aflibercept have been investigated. For example, an identical isomer of aflibercept called ziv-aflibercept has been shown to have comparable effect to aflibercept though it costs about half of bevacizumab (around $30 per dose) [18, 19].

As another option, biosimilar drugs may play an important role in facilitating community access to treatment by reducing the costs [20]. Biosimilars are the molecules that should have similar safety, efficacy, pharmacodynamics, and pharmacokinetics, compared to the reference drugs. However, due to different processes of the manufacturing of the biosimilars, they may represent various biological characteristics particularly in terms of stability of the drugs and immunogenicity [20, 21].

In brief, biosimilars are complex biological molecules that are highly similar to the reference drugs with minor differences structurally, so the efficacy and safety of the agents should be warranted by experimental and clinical studies [8, 20].

In agreement with previous studies [22], we considered at least 20% change in ERG amplitude and latency to be significant. In this study, there were no changes in ERG parameters more than 20% at each time point. The maximum change was related to the *a*-wave amplitude at day 14 in the biosimilar aflibercept group (14.6%) which was partly comparable with the reference drug group at month 1 (10.5%).

Likewise, there was no significant change in the *b*-wave amplitude during the study; the maximum change in the biosimilar aflibercept group belonged to week 4 (9.1%) which was similar with the reference drug group (10%). Also, there was no statistically significant difference in ERG parameters between the biosimilar group and the two other groups at different time points (Table 1). The mild variability of the ERG responses may be due to the technical biases, and also, it can be affected by depth of sedation. However, the results of the study revealed that biosimilar aflibercept did not affect the ERG response significantly at multiple time points' assessment, but we should keep in mind that the short-term results of the study could not be extrapolated to the long-term period, especially after multiple injections. We also evaluated the histopathology of enucleated eyes. In the biosimilar group, all the rabbits which were enucleated at 48 hours had no pathologic finding unless one that showed chronic conjunctivitis and iridocyclitis; similar finding was evident in the contralateral eye which received saline. We speculated that it may be due to the previous injury rather than the toxicity of the drug, regarding the type of the cells involved in the inflammatory response, symmetry between two eyes, and the time between injection and presentation of disease. Also, mild choroidal congestion was detected in a rabbit enucleated at week 1; interestingly, the other eye (saline) showed the similar finding. None of the other rabbits enucleated at weeks 2 and 4 showed inflammatory responses or necrosis in the histopathologic study. Notably, in the Eylea^®^ group, one rabbit showed vitritis and lymphoplasmocytic infiltration of the optic disc, which may be related to the drug injection, while the contralateral eye (saline) was normal. Intravitreal injection of the drugs can damage the eye by several mechanisms including the direct toxicity of the vehicle, inflammatory response against the immunogenic molecules, and interaction with the VEGF pathway and signalling which play a role in inflammatory responses [23].

On the basis of the ERG and histologic evaluation, the biosimilar did not show significant toxicity on the ocular tissues, particularly, the retina. However, it should be taken into account that the ERG mainly represents the photoreceptors and bipolar cell function and is not applied to the ganglion cells [24, 25]. Furthermore, the full-field ERG is representative for the mass response of the retina and is not sensitive to the localized damages. Although the histologic finding did not show any damage to the ganglion cells or other parts of the retina, there may be ultrastructural changes which cannot be detected by light microscopy [26].

This study showed the safety of intravitreal biosimilar aflibercept injection in a short-term evaluation. However, further studies are necessary to validate the safety profile and probable efficacy of this new biosimilar drug in longer follow-up durations.

## Figures and Tables

**Table 1:** Comparison of the amplitudes and latent times of rod responses from the aflibercept-treated rabbits and saline-treated rabbits and Eylea-treated rabbits.

Groups	*a*-wave amplitude	*b*-wave amplitude	*a*-wave latent time	*b*-wave latent time
48 hours				
Saline	−43.5 ± 8.8	139.4 ± 19.2	14.7 ± 0.7	35.6 ± 1.2
Eylea	−44.7 ± 5.9	120.8 ± 13.6	13.7 ± 0.6	36.1 ± 1
Aflibercept	−48.9 ± 20.3	158.6 ± 64.5	14.6 ± 0.6	37.7 ± 1.7

*P* value^*∗*^				
Aflibercept/saline	0.60	0.32	0.77	0.82
Aflibercept/Eylea	0.73	0.62	0.81	0.86

1 week				
Saline	−44 ± 8.1	149.9 ± 27.2	14.1 ± 0.6	35.1 ± 1.6
Eylea	−38.3 ± 5.8	116.5 ± 11.7	13.9 ± 0.1	32.7 ± 1.5
Aflibercept	−46.4 ± 9.9	151.4 ± 32.7	14.3 ± 0.8	36 ± 1.3

*P* value^*∗*^				
Aflibercept/saline	0.85	0.89	0.84	0.63
Aflibercept/Eylea	0.76	0.27	0.82	0.47

2 weeks				
NS	−33.6 ± 8.4	125 ± 28.3	14.3 ± 0.7	35.9 ± 1.5
Eylea	−44.5 ± 1.7	131.5 ± 22.1	13.2 ± 0.5	33.1 ± 1.5
Aflibercept	−37.8 ± 10.1	133.2 ± 40.2	14.4 ± 1.1	35.8 ± 1.4

*P* value^*∗*^				
Aflibercept/saline	0.29	0.44	0.97	0.96
Aflibercept/Eylea	0.26	0.83	0.79	0.71

4 weeks				
NS	−41.6 ± 13.3	140.3 ± 20.5	14.4 ± 0.6	37.2 ± 2.3
Eylea	−32.9 ± 8.5	105.4 ± 11.7	14.1 ± 0.1	34.3 ± 1.8
Aflibercept	−38.4 ± 20.5	140.9 ± 89.6	15.4 ± 1.1	40.2 ± 2.2

*P* value^*∗*^				
Aflibercept/saline	0.23	0.98	0.45	0.55
Aflibercept/Eylea	0.69	0.21	0.62	0.38

Data are mean ± SD ^*∗*^Mann–Whitney U-test.

**Table 2:** The amplitudes and latent times of ERG waves from the biosimilar aflibercept-treated rabbits.

Groups	*a-*wave amplitude	*b-*wave amplitude	*a-*wave latent time	*b-*wave latent time
48 hours				
Pretreatment	−50.4 ± 9.6	155.6 ± 30.1	13.1 ± 0.5	36.1 ± 0.5
Posttreatment	−48.9 ± 20.3	158.6 ± 64.5	14.6 ± 0.6	37.7 ± 1.7
Difference in %	Decrease 3%	Increase 1.9%	Increase 11.4%	Increase 4.4%

1 week				
Pretreatment	−50.4 ± 9.7	158.9 ± 24.7	14.1 ± 0.7	36.5 ± 0.6
Posttreatment	−46.4 ± 9.9	151.4 ± 32.7	14.3 ± 0.8	36 ± 1.3
Difference in %	Decrease 8%	Decrease 4.8%	Increase 1.4%	Decrease 1.4%

2 weeks				
Pretreatment	−44.3 ± 9.6	148.6 ± 27.4	14.1 ± 0.5	36.4 ± 0.8
Posttreatment	−37.8 ± 10.1	133.2 ± 40.2	14.4 ± 1.1	35.8 ± 1.4
Difference in %	Decrease 14.6%	Decrease 10.3%	Increase 2.1%	Decrease 1.7%

4 weeks				
Pretreatment	−43 ± 8.7	154.9 ± 28.8	14.1 ± 0.6	36.7 ± 0.7
Posttreatment	−38.4 ± 20.5	140.9 ± 89.6	15.4 ± 1.1	40.2 ± 2.2
Difference in %	Decrease 10.7%	Decrease 9.1%	Increase 9.2%	Increase 9.5%

Data are mean ± SD.

**Table 3:** The amplitudes and latent times of ERG waves from the Eylea-treated rabbits.

Groups	*a*-wave amplitude	*b*-wave amplitude	*a*-wave latent time	*b*-wave latent time
48 hours				
Pretreatment	−47.8 ± 3	140.1 ± 14.7	13.1 ± 0.5	37.5 ± 1.5
Posttreatment	−44.7 ± 5.9	120.8 ± 13.6	13.7 ± 0.6	36.1 ± 1
Difference in %	Decrease 6.4%	Decrease 13.8%	Increase 4.5%	Decrease 3.8%

1 week				
Pretreatment	−41.8 ± 3	137.1 ± 14.7	14.1 ± 0.5	34.1 ± 1.5
Posttreatment	−38.3 ± 5.8	116.5 ± 11.7	13.9 ± 0.1	32.7 ± 1.5
Difference in %	Decrease 8.4 %	Decrease 15.1%	Decrease 1.5%	Decrease 4.2%

2 weeks				
Pretreatment	−41.8 ± 3	126.12 ± 14.7	14.0 ± 0.5	32.4 ± 1.9
Posttreatment	−44.5 ± 1.7	131.5 ± 22.1	13.2 ± 0.5	33.1 ± 1.5
Difference in %	Increase 6.4%	Increase 4.2%	Decrease 5.8%	Increase 2.1%

4 weeks				
Pretreatment	−36.8 ± 3	117.1 ± 14.3	14.9 ± 0.8	36.7 ± 1.4
Posttreatment	−32.9 ± 8.5	105.4 ± 11.7	14.1 ± 0.1	34.3 ± 1.8
Difference in %	Decrease 10.5%	Decrease 10%	Decrease 5.4%	Decrease 6.6%

Data are mean ± SD.

## Data Availability

The ERG and histopathology data used to support the findings of this study are available from the corresponding author upon request. The authors also inserted all the ERG data in Tables 1–3.
